# T1 Mapping Quantifies Spinal Cord Compression in Patients With Various Degrees of Cervical Spinal Canal Stenosis

**DOI:** 10.3389/fneur.2020.574604

**Published:** 2020-10-30

**Authors:** Ilko L. Maier, Sabine Hofer, Eva Eggert, Katharina Schregel, Marios-Nikos Psychogios, Jens Frahm, Mathias Bähr, Jan Liman

**Affiliations:** ^1^Department of Neurology, University Medical Center Göttingen, Göttingen, Germany; ^2^Biomedizinische NMR, Max-Planck-Institut für Biophysikalische Chemie, Göttingen, Germany; ^3^Department of Neuroradiology, University Medical Center Göttingen, Göttingen, Germany; ^4^Department of Neuroradiology, Heidelberg University Hospital, Heidelberg, Germany; ^5^Department of Neuroradiology, Universitätsspital Basel, Basel, Switzerland

**Keywords:** cervical spinal canal stenosis, cervical spondylotic myelopathy, spinal cord compression, MRI, T1 mapping, T1 relaxometry

## Abstract

Age-related degeneration of the cervical spinal column is the most common cause of spinal cord lesions. T1 mapping has been shown to indicate the grade and site of spinal cord compression in low grade spinal canal stenosis (SCS). Aim of our study was to further investigate the diagnostic potential of a novel T1 mapping method at 0.75 mm resolution and 4 s acquisition time in 31 patients with various grades of degenerative cervical SCS. T1 mapping was performed in axial sections of the stenosis as well as above and below. Included subjects received standard T2-weighted MRI of the cervical spine (including SCS-grading 0-III), electrophysiological, and clinical examination. We found that patients with cervical SCS showed a significant difference in T1 relaxation times within the stenosis (727 ± 66 ms, mean ± standard deviation) in comparison to non-stenotic segments above (854 ± 104 ms, *p* < 0.001) and below (893 ± 137 ms, *p* < 0.001). There was no difference in mean T1 in non-stenotic segments in patients (*p* = 0.232) or between segments in controls (*p* = 0.272). Mean difference of the T1 relaxation times was significantly higher in grade III stenosis (234 ± 45) vs. in grade II stenosis (176 ± 45, *p* = 0.037) vs. in grade I stenosis (90 ± 87 ms, *p* = 0.010). A higher difference in T1 relaxation time was associated with a central efferent conduction deficit. In conclusion, T1 mapping may be useful as a tool for SCS quantification in all grades of SCS, including high-grade stenosis with myelopathy signal in conventional T2-weighted imaging.

## Introduction

Narrowing of the cervical spinal canal is a frequent finding in neuroimaging of the elderly. Degenerative changes, mostly causing spinal canal stenosis, affect up to 90% of the population over the age of 60 years (1). They can lead to significant clinical morbidity by causing cervical spinal canal stenosis (SCS) and, in greater extent, spinal cord compression. The onset of clinical signs of these conditions can both be insidious or sudden and appear in a wide range of symptoms, such as motoric, sensory or autonomous deficits.

The variability in the clinical presentation in patients with SCS often makes it difficult to establish the diagnosis early. However, early treatment is essential to keep track of the course of the disease and to prevent persistent deterioration (2). Further pitfalls in the diagnosis and therapy of SCS are inconsistent recommendations concerning conservative or surgical treatment [including its ideal timepoint (3)] and the diagnostic procedures this decision should be based on (4, 5).

Although clinical history and examination are the most important tools in the diagnostic workup of patients with suspected SCS, spinal neuroimaging is inevitable to confirm and further refine the diagnosis. The chosen imaging technique should not only be able to confirm the diagnosis, but also quantify its extent and exclude other differential diagnosis. To date, conventional magnetic resonance imaging (MRI), including T1- and T2-weighted imaging (T1w and T2w), is the method of choice to diagnose SCS. It allows for the evaluation of vertebrae, spinal cord, and surrounding soft tissue. T1w-hypointensities and T2w-hyperintensities help to indicate microstructural abnormalities of the spinal cord in advanced and late stages of SCS with severe and often long-term compression (6). In contrast, in early and mild stages of SCS no changes in T1w and T2w spinal cord signal is visible, often despite the presence of advanced neurological deficits. Furthermore, spinal cord evaluation with conventional MRI is limited by operator-dependency and associated inter-rater variability (7). Intermittent posture dependent spinal cord compression in low grade SCS cannot be visualized and signal intensity changes in the spinal cord are usually only seen in advanced stages of SCS, in which surgical therapy in most cases doesn't lead to significant clinical improvements (7–9).

There are numerous developments in imaging of SCS to improve the diagnostic process, such as recent studies on dynamic cervical MRI (10), as well as quantitative MRI including ultra high-field structural MRI (11). To date, these imaging techniques are not widely available and require prolonged imaging protocols. In view of the development of an aging population with rising numbers of SCS patients, a fast and reliable imaging technique is needed to diagnose and quantify spinal cord compression in various degrees of SCS.

In order to develop a sensitive and reproducible imaging biomarker that allows for early diagnosis, quantification of spinal cord compression and (ultimately) outcome prediction, a recent study indicated promising diagnostic potential of a novel high-resolution single-shot T1 mapping technique at 3T (12). In this study, even clinically slightly affected patients without spinal cord compression on conventional MRI (low grade SCS with narrowed spinal canal) showed decreased T1 relaxation times within the maximum of the SCS. Moreover, the grade of the SCS was associated with the difference in the T1 relaxation time and therefore with the extent of spinal cord compression.

The aim of the present study was to further evaluate the potential of quantitative T1 mapping in the diagnosis of cervical SCS, by including patients with severe SCS and prolonged spinal cord compression as well as by using a 1.5 T MRI system, which is frequently used in the routine diagnostic workup of SCS.

## Materials and Methods

### Subjects

In this prospective, single center study, 41 patients (31 patients with SCS and 10 controls) were recruited in the Department of Neurology of the University Medical Center Göttingen. The 31 patients had been diagnosed with cervical SCS using standard T1w and T2w MRI prior inclusion in the study. Patients with other diseases affecting the integrity- or causing compression of the spinal cord (like tumors, infection or trauma) were excluded. Four patients without clinical or imaging findings suggestive for cervical SCS or other diseases affecting the spinal cord were included as healthy controls and scanned at 1.5 T. T1 mapping was performed in the Department of Neuroradiology of the University Medical Center Göttingen.

Exclusion criteria were regular contraindications for MRI, incapacity to give informed consent and inability to tolerate supine position for the estimated imaging-time.

All subjects were allocated to four grades of cervical SCS based on a grading system developed by Kang et al. (13), which differentiates 4 grades: in grade 0 no spinal canal stenosis is visible, in grade I the spinal canal is narrowed without compression of the spinal cord, in grade II the spinal cord is deformed and in grade III the spinal cord is compressed with additional increased signal intensity near the compressed level in T2w images.

The study was approved by the ethics committee of the University Medical Center Göttingen (6/6/17). All subjects gave written informed consent. This study was in consent with the Declaration of Helsinki.

### Clinical and Electrophysiological Data

Each subject, patients and controls, was evaluated using a numeric rating scale for pain (NRS) with the endpoints 0 (no pain) and 10 (pain as severe as imaginable), the Japanese Orthopedic Association (JOA) scale (14) adapted to European conditions (15) ranging from 0 (severe deficits) to 17 points (normal function) and the Grip and Release Test (16). NAS, JOA, and Grip and Release test of each subject were acquired by the same examiner. Electrophysiological studies were performed as being indicated by the treating neurologist including sensory-evoked (SEP) and motor-evoked (MEP) potentials. In cases in which these examinations were not requested by the treating neurologist and were not available from previous in-hospital or out-patient visits, the patient was asked for permission to perform SEPs and MEPs.

### T1 Mapping

MRI studies were conducted at 1.5 T (Magnetom Avanto Fit, Siemens Healthcare Erlangen, Germany) using a combined 20 channel head/neck coil. Anatomical images were based on a T2w fast spin-echo sequence with in-plane resolution of 0.7 mm and a slice thickness of 3 mm (repetition time TR = 4,280 ms, echo time TE = 89 ms, flip angle 120°). In patients, single-slice T1 mapping was performed in three sections in the center of the SCS as well as above and below the stenosis with a systematically set minimum distance of one segment. The maximum of the SCS was identified on sagittal T2w images and reviewed by an experienced neuroradiologist. In cases of multiple SCS the location of maximal spinal canal narrowing and spinal cord compression with the highest grade according to the classification of Kang et al. was selected (13). To quantify the extend of the SCS, we calculated a ratio of the midsagittal diameter of the spinal canal at the compression site divided by the average diameter of the spinal canal at the closest non-stenotic regions above and below (for an example see Supplementary Figure 1).

In healthy controls, T1 mapping was performed at six locations including the C2–C7 segments of the cervical spinal column. All sections of the T1 mapping were planned using sagittal T2w images and standard localizer sequences.

T1 mapping of the spinal column was performed at 0.75 mm in-plane resolution and 6 mm slice thickness in multiple axial sections perpendicular to the spinal cord. In brief, the method is based on a single inversion-recovery experiment with a leading slice-selective 180° inversion pulse, a highly undersampled radial gradient-echo readout and a non-linear inverse image reconstruction technique, for details and validation see (17). Briefly, the method employs a low-flip angle gradient-echo sequence (TR = 3.32 ms, TE = 2.30 ms, flip angle 6°) with a golden-angle radial trajectory (angle = 17°) and radiofrequency spoiling by random phase alterations (18). In order to optimize computational speed, binning of the data involved 21 spokes per frame and resulted in a temporal resolution of 70 ms for sampling the inversion-recovery process. The acquisition of a total of 57 images then yielded a measuring time of 4 s per T1 map. The method is part of a larger software package which is available for Siemens MRI systems, preferably at 3 T. It requires a license from the Max-Planck-Society and the integration of a GPU-based computer into the pipeline of the MRI system in order to run the reconstruction algorithm.

Immediately after completion of data acquisition, maps of T1 relaxation times were automatically calculated and displayed by the MRI system. The values are obtained by a pixelwise fitting of the exponential signal model to the set of reconstructed serial images. The parametric results are the equilibrium magnetization M0, the steady-state magnetization Mss and the effective relaxation rate 1/T1^*^ yielding T1 = T1^*^ ((Mss – M0)/Mss – 1) (19).

Mean T1 values of the spinal cord were obtained by manually drawing a conservative region-of-interest (ROI) of the myelon using both grayscale T1 maps without artificial color borders and corresponding T2w anatomical images. Color-coded T1 maps are only used for improved visualization. It should be noted that the high spatial resolution of the T1 maps served to minimize partial volume effects with cerebrospinal fluid which has a much longer T1 value and is easily distinguishable from spinal cord and bony structures (low signal). Data analysis and ROI definition were performed using Fiji (Fiji Is Just ImageJ), an open source image processing package based on ImageJ (20).

### Statistical Analysis

Statistical analysis was performed using SPSS 21 (IBM SPSS Statistics, Armonk, NY, USA). Baseline characteristics of all patients and controls are shown as mean ± standard deviation (SD), if normally distributed, and as median with interquartile range (IQR), if not. T1 relaxation times were compared using one-way ANOVA with repeated measurements and *post-hoc* Bonferroni correction for multiple comparisons at a threshold of *p* < 0.05. Correlations between clinical or radiological scores/measurements and T1 relaxation times were determined by a bivariate Pearson-correlation. Inter-Rater Reliability has been determined using the Intraclass Correlation Coefficient. *P*-values below 0.05 were considered statistically significant.

## Results

### Patient Characteristics

Mean age of the 31 patients with cervical SCS was 68 ± 11 years (56 ± 5 years for controls). Nineteen (61.3%) patients were male (4 male controls) (Table 1). The maximum of the cervical SCS was most frequently found in the C5/C6 segment (15/31 patients) followed by the segments C3/4, C4/5, and C6/7 (5/31 patients each). Most patients had a grade I cervical SCS (16/31) followed by grade II stenosis (10/31) and grade III stenosis (5/31).

**Table 1:** Baseline characteristics of patients with cervical SCS and controls.

	**Patients with cervical SCS (*n =* 31)**	**Controls (*n =* 4)**
Age (mean ± SD)	68 ± 10	56 ± 5
Sex male (n, %)	19 (61.3)	4 (100)
Height (mean cm ± SD)	173 ± 8	177 ± 5
Body weight (mean kg ± SD)	76 ± 12	97 ± 22
**Height of SCS**		
C2/3 (*n*, %)	1 (3.2)	n.a.
C3/4 (*n*, %)	5 (16.1)	n.a.
C4/5 (*n*, %)	5 (16.1)	n.a.
C5/6 (*n*, %)	15 (48.4)	n.a.
C6/7 (*n*, %)	5 (16.1)	n.a.
C7/Th1 (*n*, %)	0 (0)	n.a.
Grade of SCS median (IQR)	1 (1-2)	0 (0)
Grade 0 (*n*, %)	0 (0)	4 (100)
Grade I (*n*, %)	16 (51.6)	n.a.
Grade II (*n*, %)	10 (32.3)	n.a.
Grade III (*n*, %)	5 (16.1)	n.a.
History of spine surgery (*n*, %)	4 (12.9)	0 (0)
**Clinical scores**		
JOA (median, IQR)	15.0 (12,25–16)	16.5 (15.9–17)
Grip and release test (median of number of grip and releases, IQR)	18.0 (15.3–20.8)	25.5 (21.75–26.25)
Pain NRS (median, IQR)	0 (0–0)	0 (0–0)

*SCS, spinal canal stenosis; SD, standard deviation; IQR, interquartile range; NRS, numeric rating scale*.

Median JOA was lower in patients with SCS [15 points (IQR, 12.25–16)] compared to controls [16.5 points (IQR, 15.9–17)]. The median pain-NRS was 0 points in both groups. Number of grip and releases in 10 s. was 18 (IQR, 15.3–20.8) in SCS patients and lower compared to healthy controls [25.5 (IQR, 21.75–26.25)].

### T1 Relaxation Times

**Figure 1:**
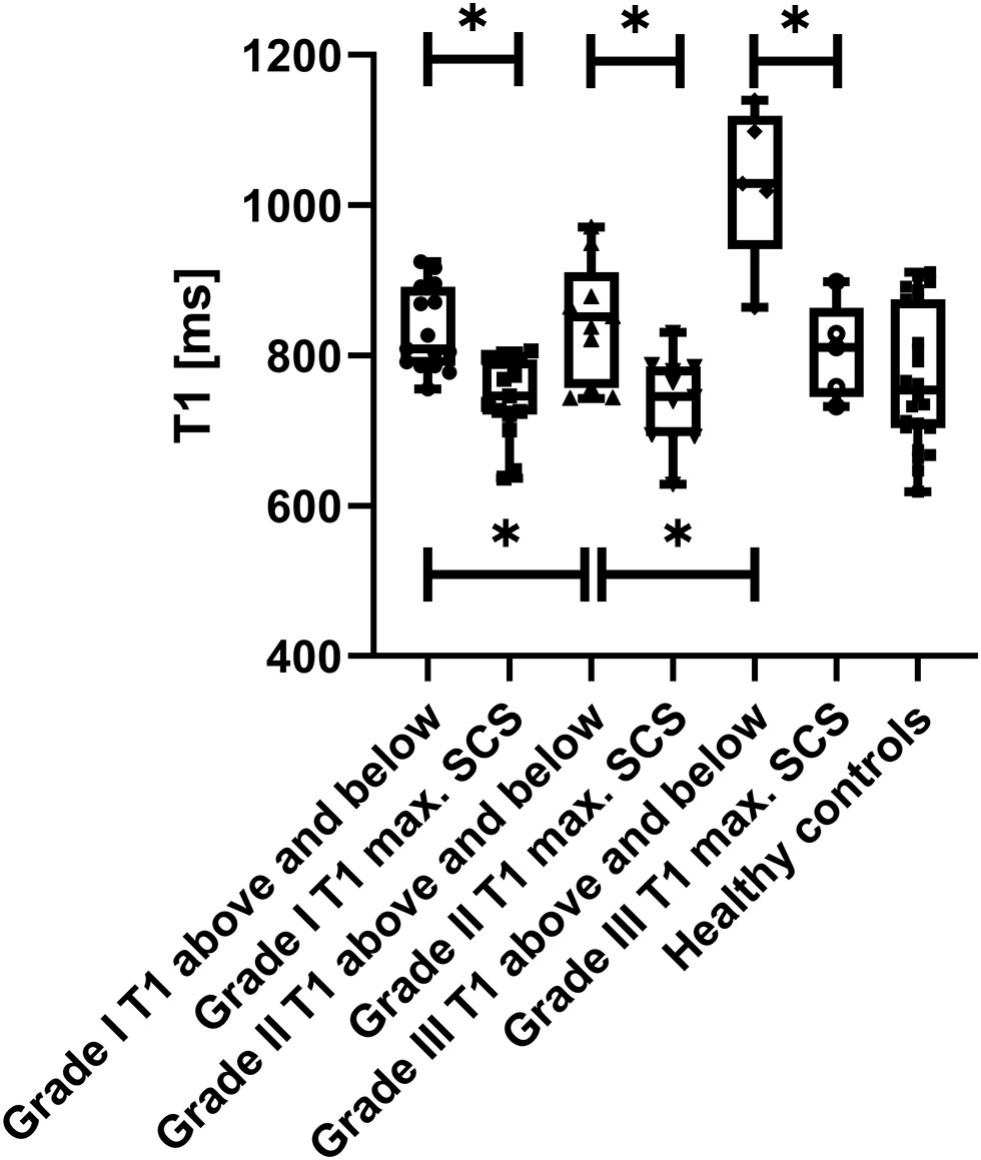
Box-and-Whisker Plots of absolute T1 relaxation times above and below the spinal canal stenosis, T1 relaxation times within the maximum of the stenosis as well as mean T1 relaxation times along the spinal cord (C2–C7) in healthy controls are visualized. Note the decrease in T1-relaxation times within the stenosis compared to above and below, which is highest in the grade III SCS group. Also note the gradual increase of the mean T1 relaxation time above and below with the grade of the stenosis. Mean T1 relaxation times in heathy controls (1.5 T) are lower compared to non-stenotic regions in SCS patients, indicating pathophysiological involvement of these regions. *statistically significant at a threshold of *p* < 0.05.

**Figure 2:**
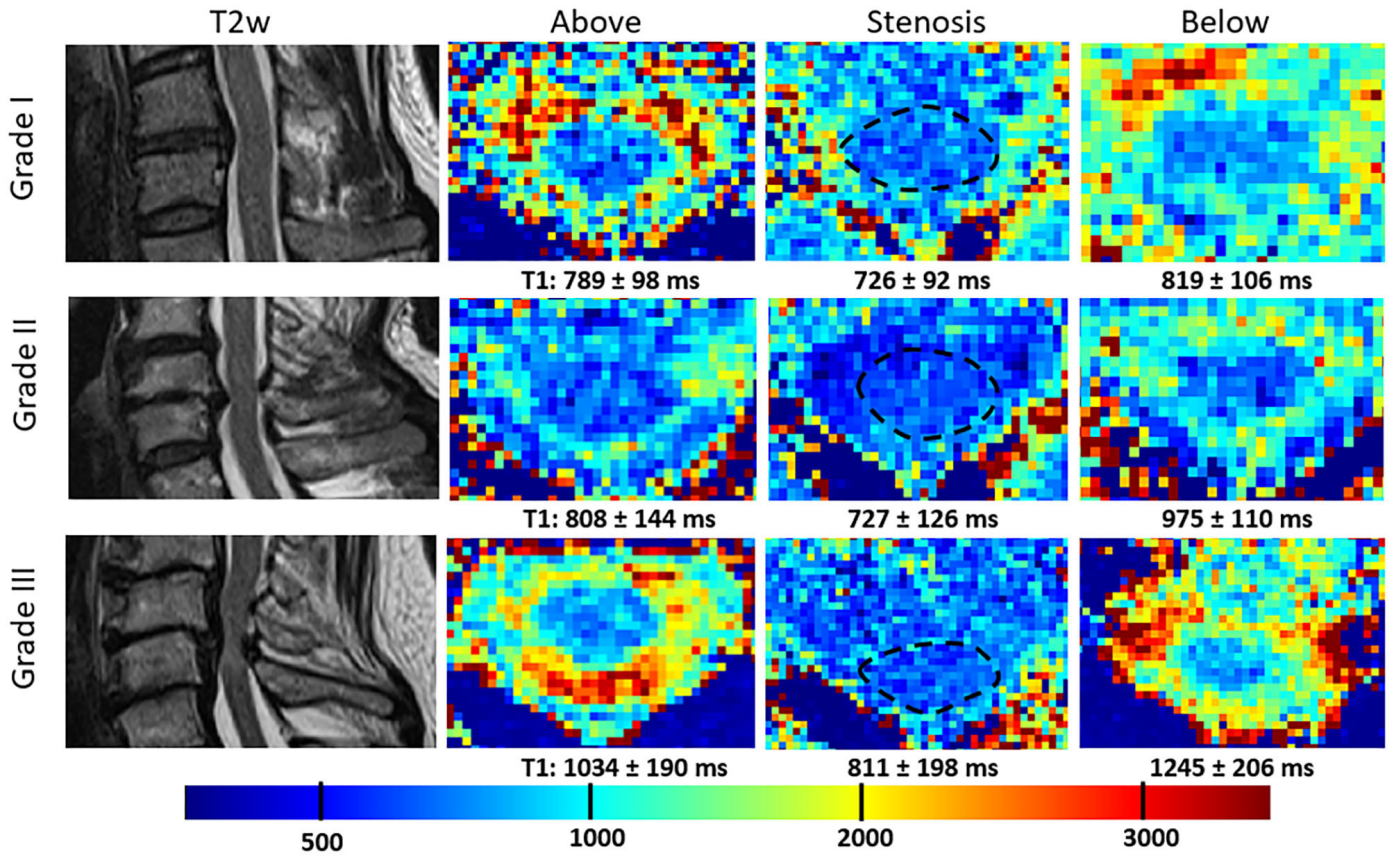
Representative sagittal T2w images and T1 maps of patients with grade I-III cervical SCS within, above and below its maximum. Note the decrease in T1 relaxation time within the stenosis in all three grades and the overall increased T1 relaxation time in grade III stenosis within as well as above and below the stenosis.

Overall T1 relaxation times within the stenosis differed significantly in all grades [727 ± 66 vs. 854 ± 104 ms above (*p* < 0.001) and 893 ± 137 ms below (*p* < 0.001)] as well as in the individual SCS-grade groups I-III compared to adjacent, non-stenotic parts of the spinal cord (Table 2, Figure 1). Moreover, there was a grade dependent difference in T1 relaxation times from grade I to grade III in regions above and below the compressed spinal cord (Figure 1). Both changes are visualized in Figure 2, showing T1 maps of three representative cases with a grade I-III SCS.

**Table 2:** Comparison of absolute T1 relaxation times in different grades of cervical spinal canal stenosis.

	**Cervical Spinal Canal Stenosis**				
	**Grade I**	**Grade II**	**Grade III**	***p*-value grade I vs. III**	***p*-value grade II vs. III**
T1 (ms ± SD) in stenosis	726 ± 58	693 ± 48	797 ± 72	0.081	0.008
T1 (ms ± SD) above stenosis	818 ± 80	835 ± 80	984 ± 110	0.003	0.012
T1 (ms ± SD) below stenosis	814 ± 97	903 ± 90	1,076 ± 138	<0.001	0.015
T1 (ms ± SD) in non-stenotic spinal cord (mean T1 above and below)	816 ± 58	869 ± 73	1,013 ± 105	<0.001	0.001

*SD, standard deviation*.

**Figure 3:**
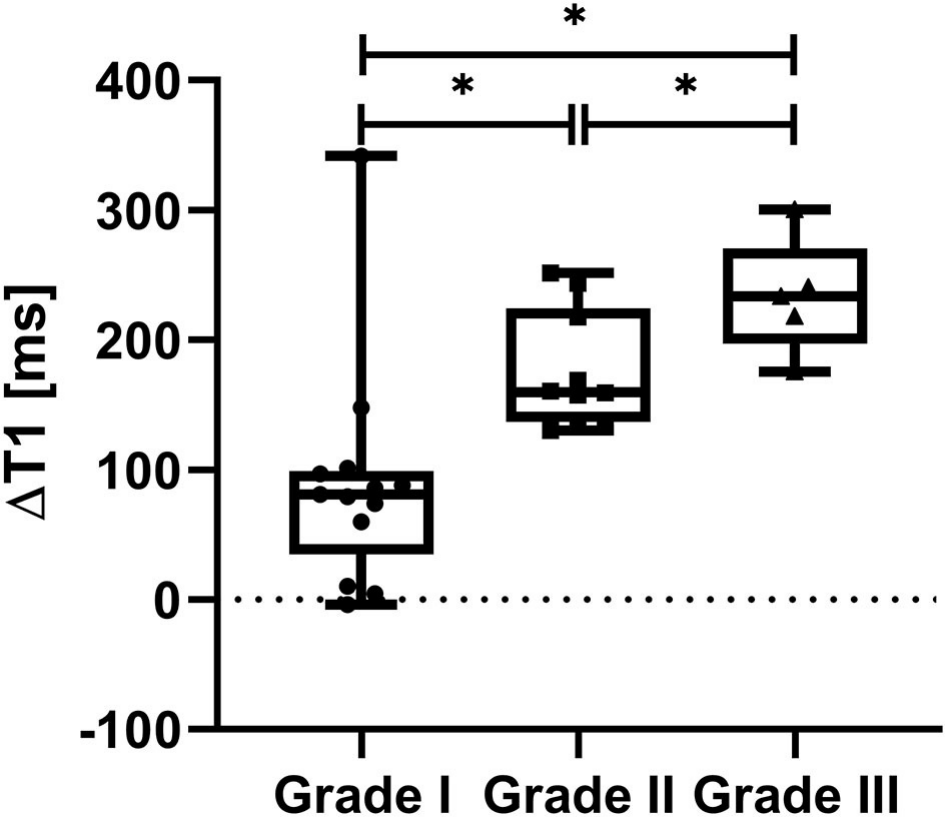
Difference in T1 relaxation times in grade I-III stenosis. Note the increase in the difference of T1 relaxation times, which is lowest in grade I and highest in grade III stenosis. *statistically significant at a threshold of *p* < 0.05.

The mean difference of the T1 relaxation times (mean T1 above and below minus T1 within the stenosis) was significantly higher in grade III stenosis (234 ± 45 ms) vs. in grade II stenosis (176 ± 45, *p* = 0.037) vs. in grade I stenosis (90 ± 87 ms; *p* = 0.010) (Figure 3). There was a significant negative correlation between the maximum extend of the SCS (midsagittal diameter of the spinal canal at the compression site divided by the average diameter of the spinal canal above and below) and the difference in T1 relaxation time (*r* = −0.534, *p* = 0.002).

There were no differences in absolute T1 relaxation times between non-stenotic segments of the spinal cord above and below the stenosis in all three SCS groups (*p* = 0.232) as well as no significant variation concerning the T1 relaxation times in healthy controls in the C2–C7 segments at 1.5 T (*n* = 4, mean T1 relaxation time of all segments 771 ± 97 ms; *p* = 0.272). No differences in absolute T1 relaxation times between non-stenotic spinal cord segments above and below the stenosis and healthy controls, nor differences between stenosis and healthy controls, could be noticed.

Table 2 shows the absolute T1 relaxation times in different grades of SCS within the stenosis as well as in the non-stenotic spinal cord above and below. Patients with a grade III stenosis showed higher T1 relaxation times within the stenosis (grade I vs. III: *p* = 0.081; grade II vs. III: *p* = 0.008) as well as higher T1 relaxation times in the non-stenotic spinal cord (grade I vs. III: *p* < 0.001; grade II vs. III: *p* = 0.001) compared to patients with a lower grade SCS (Table 2). There was no significant difference between absolute T1 relaxation times in patients with grade I and grade II SCS (grade I: 726 ± 58 vs. grade II: 693 ± 48; *p* = 0.156).

T1 relaxation times were analyzed by two independent raters showing an excellent intra class correlation for T1 values above/below the stenosis (0.936, *p* < 0.001) and good intra class correlation for T1 values in the stenosis (0.788, *p* < 0.001).

### Clinical and Electrophysiological Findings

Both SEP and MEP investigation was available from 23 (74.2%) patients (Table 3). From these 23 patients, 10 (43.5%) showed a central efferent conduction deficit and 16 (69.6%) showed a central afferent conduction deficit. Patients with a central efferent conduction deficit showed a non-significant trend toward a higher difference in T1 relaxation times within the stenosis (196 ± 89 vs. 127 ± 81 ms; *p* = 0.056) and a non-significant trend toward a lower number of repeats in the grip-and-release test (16 ± 3 vs. 19 ± 4; *p* = 0.129), while there were no differences in JOA score or absolute T1 values in both groups. In patients with and without central afferent conduction deficits, there were no difference in absolute T1 values, change in T1 relaxation time and clinical tests. A subanalysis of the T1 values in dorsal and ventral parts of the spinal cord as well as of SCS with dorsal and ventral compression resulted in no significant changes or an improved correlation between T1 relaxation times and electrophysiology/clinical signs of spinal cord compression (data not shown).

**Table 3:** T1 relaxation times in patients with and without central conduction deficits (*n* = 23).

	**Central efferent conduction deficit (*n =* 10)**	**No central efferent conduction deficit (*n =* 13)**	***p*-value**	**Central afferent conduction deficit (*n =* 16)**	**No central afferent conduction deficit (*n =* 7)**	***p*-value**
T1 in stenosis (ms ± SD)	736 ± 89	735 ± 39	0.997	731 ± 71	704 ± 71	0.364
ΔT1 (ms ± SD)	196 ± 89	127 ± 80	0.056	135 ± 86	164 ± 73	0.448
Grade of stenosis (median, IQR)	2 (1–3)	1 (1–2)	0.110	1 (1–2)	2 (1–3)	0.557
JOA (median score, IQR)	14.2 ± 2	14.9 ± 2	0.422	14 ± 2	16 ± 1	0.004
Grip and release test (median, IQR)	16 ± 3	19 ± 4	0.129	17 ± 3	20 ± 4	0.066

## Discussion

In this confirmatory study, we were able to reproduce findings from an earlier pilot study, in which a difference in T1 relaxation times of the spinal cord within the maximum of a cervical SCS have been found (12). In detail, the present study expands our previous findings as follows: First, we again found lower absolute T1 relaxation times in all grades of SCS and in addition were able to demonstrate a grade dependent difference in T1 relaxation times at the lower field strength of 1.5 T, even though the T1 maps acquired at this lower field strength had lower in-plane resolution and higher in-ROI SD values. Nevertheless, we could demonstrate excellent/good inter-rater reliabilities, indicating reproducibility of T1 relaxation time measurements despite manual delineation of the spinal cord. However, in our study only one scan per patient/control had been performed and therefore inter-session reproducibility could not be demonstrated. Second, patients with grade III stenosis, defined as spinal cord compressions showing T2w-hyperintense spinal cord lesions were included in this study, while such patients did not participate in the initial pilot study. Grade III SCS has been shown to be associated with unfavorable clinical outcomes and decreased response to decompressive surgery (9, 15, 21). In these patients, the absolute T1 values within the stenosis as well as in non-stenotic regions of the spinal cord were higher compared to patients with grade II stenosis, while the difference in T1 relaxation time were significantly higher. This indicates that (1) difference in T1 relaxation time is the most sensitive marker for spinal cord compression compared to absolute T1 values and (2) edema or gliosis, both possible correlates of T2w-hyperintensities in SCS, are not only present within the stenosis in grade III stenosis (increasing the T1 relaxation time), but also influence the segments above and below the stenosis (and therefore increasing their T1 relaxation times). This finding indicates that, in severe spinal cord compression, the T1 relaxation time is altered within the stenosis, but also in adjacent segments of the spinal cord, likely to be involved in the pathophysiological process of the disease (e.g., edema formation or demyelinisation). This again indicates a huge advantage of T1 mapping compared to conventional T1w and T2w MRI, as these changes cannot be visualized by these techniques and could explain the common inconsistence between clinical findings and imaging. Another advantage of the T1 mapping technique is that it's very fast and therefore is less susceptible for motion artifacts compared to other imaging techniques like diffusion tensor imaging, which could be used in combination to provide complementary information in future studies. In addition, previously published T1-mapping techniques applied in the human spinal cord are characterized by long acquisition times (22–24). Therefore, our T1-mapping technique has potential to be integrated in routine MRI-protocols with no significant prolongation of the scanning time and being robust concerning motion artifacts. Third, compared to the pilot study, we could double the number of included patients, increasing statistical power.

Overall, the association between T1 values, clinical, and electrophysiological parameters were not convincing, which is similar to the results of our previous study. This may be explained by the fact that spinal canal narrowing and spinal cord compression caused by degenerative changes are gradual and progress over a long period of time, inducing various compensatory mechanisms (25). Moreover, all included patients were not scheduled for surgery, but handled with a conservative treatment approach, which is likely to be recommended in patients complaining about mild symptoms only and with a long-term stable clinical course.

The predictive value of the drop in T1 relaxation times in various degrees of SCS for the success of surgery and deterioration in the further course of the disease to date is unclear. An ongoing study in our center currently focusses on changes of the T1 relaxation time in the maximum of the spinal cord compression in patients with surgical decompression and conservative therapy. With this study, we aim to confirm both our previous (12) and present findings derived from cross-sectional measurements as well as aim to investigate longitudinal changes of T1 relaxation times within SCS over time.

Limitations of this study include those inherent to a small sample size and a cross-sectional study design. The small sample size also includes the low number of control subjects, of which the recruitment-difficulties have already been discussed in our previous study (12). However, neither in the study of Battiston et al. (22), originally describing T1 mapping of the human spinal cord, nor in our previous and present study significant variation of T1 relaxation times were found. Consequently, we assume that variation of T1 relaxation times of the spinal cords are likely to be represent involvement in disease processes rather than representing physiological variations. However, recent studies performed T1 mapping at 3T and the reproducibility of the T1 technique at 1.5 T needs to be fully quantified in future studies. As we did not expect changes of the T1 relaxation times in non-stenotic parts of the spinal cord in SCS patients, we only performed one “control” measurement above and below the stenosis with a systematically set distance of one segment. Therefore, a possible “gradient” of T1 values cannot be ruled out by our study and needs to be investigated by further measurements in the segments above and below the stenosis. Moreover, an age-dependency of T1 relaxation times of the healthy spinal cord can't be ruled out as only a low number of healthy controls could be included in this study and age has not been considered as covariate in the statistical analysis. Further studies investigating disease-related changes in T1 relaxation times of the human central nervous system should include a high number of healthy controls of different age cohorts for adequate comparison with patients and to deal with potential bias induced by age. This assumption underlines the importance of the inclusion of age-matched controls in further studies.

From a methodological point of view, in future studies axial T2w images of the maximum stenosis should be analyzed in detail to investigate the involvement of district motor- or sensory fiber tracks and to allow for a higher accuracy of spinal cord delineation within the stenosis, which both also might lead to a clearer association between T1 relaxation times and clinical/electrophysiological findings. In the present study, the latter association is also limited by missing examinations in around 25% of cases. Also, some bias in patient selection cannot be excluded as all participants were from a cohort managed with a conservative approach. Moreover, raters of T1 relaxation times were not blinded to clinical data and results from conventional MRI. Overall, the present study lacks patients with severe neurologic deficits associated with SCS. The major reason for this is the inclusion of patients with conservative management only and therefore, with the present findings, conclusions can only be drawn in this patient group.

## Conclusion

In conclusion, we provide additional evidence for the usefulness of T1 mapping in patients with degenerative SCS to allow investigating normal appearing tissue on T1w or T2w imaging, thus providing additional characterization of tissue impairment. Based on our results, a future study is needed in which this fast, non-invasive, and reproducible imaging technique must be applied in SCS-patients before and after decompressive surgery to gain further insights in its predictive value and to investigate longitudinal changes in the T1 relaxation time in surgically vs. conservatively managed patients.

## Data Availability Statement

The raw data supporting the conclusions of this article will be made available by the authors upon reasonable request.

## Ethics Statement

The study was approved by the ethics committee of the University Medical Center Göttingen (6/6/17). All subjects gave written informed consent. The patients/participants provided their written informed consent to participate in this study.

## Author Contributions

IM designed the study and was involved in the acquisition of the data, analyzed raw data, performed statistics, drafted and finalized the manuscript, and approved the manuscript before submission. SH designed the study and was involved in the acquisition of the data, analyzed raw data, contributed to the manuscript, and approved the manuscript before submission. EE and KS was involved in the acquisition of the data, contributed to the manuscript, and approved the manuscript before submission. MN-P contributed to the manuscript and approved the manuscript before submission. JF designed the study, finalized the manuscript, and approved the manuscript before submission. MB initiated and designed the study, contributed to the manuscript, and approved the manuscript before submission. JL designed the study, contributed to the manuscript, and approved the manuscript before submission. All authors contributed to the article and approved the submitted version.

## Conflict of Interest

M-NP received speakers' honoraria from Siemens Healthineers. The remaining authors declare that the research was conducted in the absence of any commercial or financial relationships that could be construed as a potential conflict of interest.
