# Supplementary material for: T1 Mapping Quantifies Spinal Cord Compression in Patients With Various Degrees of Cervical Spinal Canal Stenosis
Source: Front Neurol. 2020 Oct 30;11:574604. doi: 10.3389/fneur.2020.574604 (PMC7662110; doi:10.3389/fneur.2020.574604)
Supplement: Supplementary file 1 [file Data_Sheet_1.PDF]

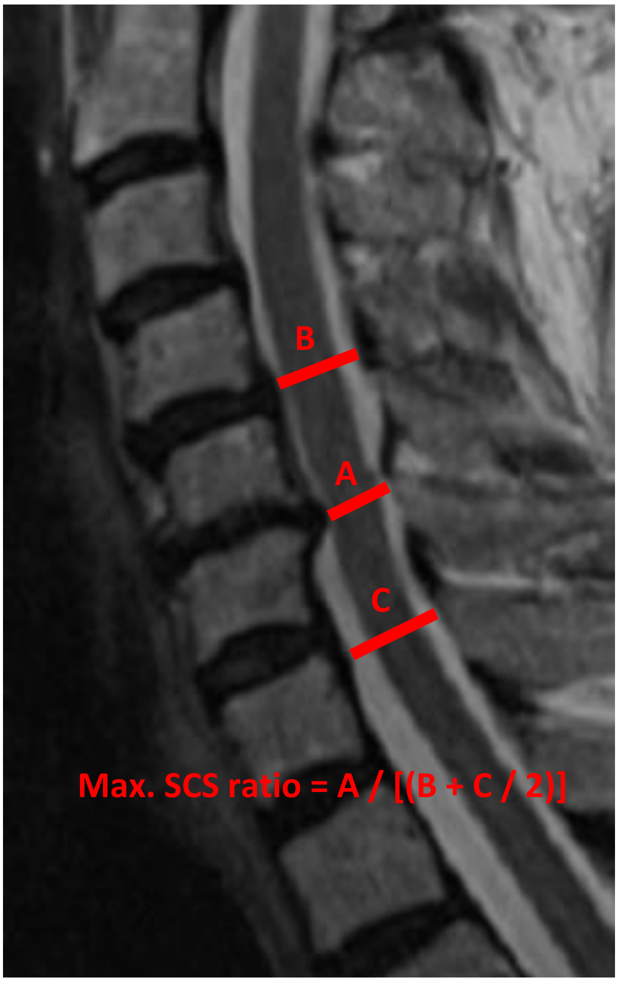

Supplementary Fig. 1: To quantify the extend of the SCS, we calculated a ratio of the midsagittal diameter of the spinal canal at the compression site divided by the average diameter of the spinal canal at the closest noncompressed regions above and below.
